# 4‐Aminothiophenol Photodimerization Without Plasmons**

**DOI:** 10.1002/anie.202205013

**Published:** 2022-05-19

**Authors:** Ivano Alessandri

**Affiliations:** ^1^ Sustainable Chemistry and Materials Group Department of Information Engineering University of Brescia Italy; ^2^ INSTM Consorzio Nazionale per la Scienza e Tecnologia dei Materiali RU Brescia via Branze 38 25123 Brescia Italy; ^3^ INO-CNR RU Brescia via Branze 43 25123 Brescia Italy

**Keywords:** DMAB, PATP, Photodimerization, Surface-Enhanced Raman Spectroscopy, Singlet Oxygen

## Abstract

The photodimerization of 4‐aminothiophenol (PATP) into 4,4′‐dimercaptobenzene (DMAB) has been extensively utilized as a paradigm reaction to probe the role of surface plasmons in nanoparticle‐mediated light‐driven processes. Here I report the first observation of the PATP‐to‐DMAB photoreaction in the absence of any plasmonic mediators. The reaction was observed to occur with different kinetics either for PATP adsorbed on non‐plasmonic nanoparticles (TiO_2_, ZnO, SiO_2_) or deposited as macroscopic droplets. Confocal microRaman spectroscopy enabled to investigate the reaction progress in different plasmon‐free contexts, either aerobic or anaerobic, suggesting a new interpretation of the photodimerization process, based on direct laser‐induced activation of singlet oxygen species. These results provide new insights in light‐driven redox processes, elucidating the role of sample morphology, light and oxygen.

## Graphical abstract

The photodimerization of 4‐aminothiophenol (PATP) into 4,4′‐dimercaptobenzene (DMAB) is demonstrated to occur in the absence of any plasmonic mediators, previously considered a paradigm for driving this type of photo‐oxidation. An alternative pathway, based on direct activation of singlet oxygen, is demonstrated. These results provide new insights in light‐driven redox processes, elucidating the role of sample morphology, light and oxygen.

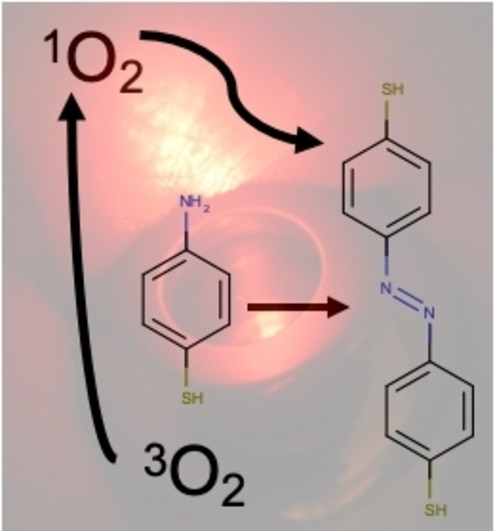

## Introduction

The photodimerization of 4‐aminothiophenol (PATP) or 4‐nitrothiophenol (PNTP) into 4,4′‐dimercaptoazobenzene (DMAB) has attracted a lot of attention in the scientific community, representing a continuous source of vibrant debates. In a seminal paper, dating back to 1994, Osawa et al. reported the differences between the Surface Enhanced Raman spectra (SERS) of PATP adsorbed on silver nanoparticles and those of the bulk counterpart.^[1]^ For many years this remarkable difference has been ascribed to the so‐called “chemical enhancement”, resulting from charge transfer from metal to the adsorbed molecule. This effect was though to selectively enhance the non‐totally symmetric (b2) modes of PATP. Later, other studies combining SERS and mass spectrometry demonstrated that the spectral differences are actually due to the formation of DMAB.^[2]^

Since then, the PATP‐to‐DMAB photodimerization has been thoroughly investigated as a model reaction to test the capability of plasmonic systems to assist light‐driven reactions. In this context, another debate on the reaction mechanism is still running. On one hand, hot carriers (electrons, in the case of PNTP photo‐reduction or holes, in the case of PATP photo‐oxidation) have been invoked to assist photodimerization by either direct charge injection into the LUMO (hot electrons) or from the HOMO (hot holes) of the adsorbed molecules.^[3]^

However, recent studies pointed out the photothermal origin of the photoreaction. In particular, plasmonic heating has been indicated to play a key role,[^4^, ^11^] whereas pure thermal heating is not effective in driving photodimerization.^[10]^ This phenomenon is quite surprising, as the overall contribution of thermal energy should not be dependent on the heating source.

In spite of different models and several contradictions, all the studies agree in considering the presence of plasmonic nanoparticles as mandatory for driving the photoreaction.^[5]^ More generally, even when the reaction was run by applying an external voltage or by adding oxidants (AgNO_3_, FeCl_3_, H_2_O_2_), metal nanoparticles have always been present.[^3^, ^6^]

Here, I unexpectedly discovered that PATP can undergo photodimerization in the absence of any metal mediators, by direct irradiation with a low power 633 nm laser (1.96 eV). This result is quite surprising, since past experiments reported the impossibility to directly convert PATP into DMAB by direct irradiation with visible lasers (633, 532, 457 nm).^[7]^ However, in this work I observed that this reaction can be not only triggered, but also controlled, by modifying size and morphology of the sample under irradiation. A possible model, based on the laser‐induced excitation of adsorbed molecular oxygen to singlet oxygen states is derived from confocal Raman microscopy experiments. Most of the unclear aspects of experimental data obtained for plasmonic systems can also be disentangled through this approach.

## Results and Discussion

**Figure 1:**
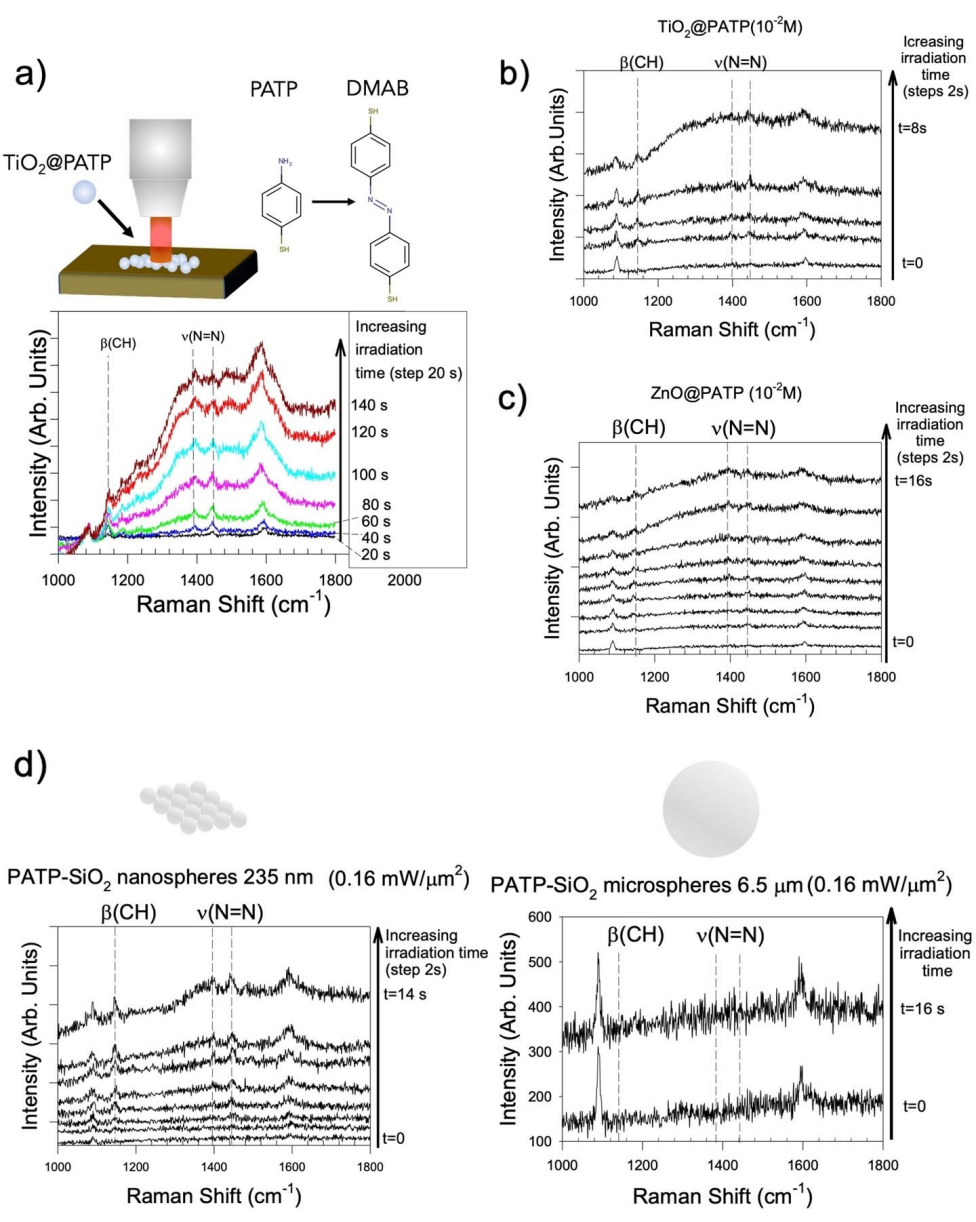
a) Evolution of PATP photo‐oxidation on P25‐TiO_2_ nanoparticles induced by laser irradiation at 633 nm. The power was reduced to 0.16 mW μm^−2^ to follow the formation of DMAB species, indicated by the β(CH) and ν(N=N) Raman modes. Each spectrum was acquired for 20 s at the time interval indicated in the label; b) the same experiment with higher concentration of PATP solution (10^−2^ M); c) same experiment of b, using ZnO nanoparticles (average size <100 nm) instead of TiO_2_; d) comparison between monodisperse nano‐ and micron‐sized SiO_2_ spheres soaked with PATP 10^−2^ M molar solution. The individual Raman spectra for experiments with 6.5 μm spheres in the interval *t*=2–14 s have been omitted for clarity and shown in Supporting Information (S5).

The first proof of direct, plasmon‐free photoconversion of PATP into DMAB was obtained unintentionally during the microRaman characterization of commercial TiO_2_ (Aeroxide® P‐25) powders previously soaked in a 10^−4^ M PATP ethanol solution for 30 minutes. Direct irradiation with a 633 nm laser focused through a 50X microscope objective (Numerical Aperture, N. A.: 0.5) with a power of 1.6 mW μm^−2^, corresponding to 5×10^15^ photons μm^−2^ s^−1^, resulted in a very fast (<1 s) degradation of the PATP coating (Supporting Information, Figure S1). By reducing the laser power to 0.16 mW μm^−2^ (5×10^14^ photons μm^−2^ s^−1^) the reaction can be controlled, and its progress is shown in Figure 1a.

We note that after 20 s new Raman modes appear at 1142, 1395 and 1443 cm^−1^ and progressively increase their intensity. According to previous studies, these signals are associated to the β(CH) and ν(N=N) modes of DMAB.^[8]^ The same evolution of the Raman signal is observed when laser irradiation power was decreased to 16 μW μm^−2^ (Figure S2). In this case the dimerization is slower, which indicates a direct dependence of reaction kinetics on the photon flux. On the other hand, increasing the concentration of PATP solution to 10^−2^ M results in faster dimerization kinetics, as shown in Figure 1b. The observation of photodimerization induced at 633 nm in the absence of any plasmonic mediators was unexpected, at least on the basis of the results reported in literature. For example, Zhang et al. did not observe any dimerization at 633 nm and concluded that the energy is not sufficient for triggering the azo‐coupling reaction.^[7]^ Thus, the first hypothesis is that, in our case, TiO_2_ could be directly involved in assisting photodimerization.

However, photodimerization was observed even with other non‐plasmonic substrates, such as ZnO (Figure 1c) or SiO_2_ nanoparticles (Figure 1d). Moreover, Figure S3 shows that the same PATP solution deposited on planar TiO_2_ thin films does not undergo any photodimerization under the same conditions of the experiment reported in Figure 1a, while by increasing the irradiation power to the pristine value of 1.6 mW μm^−2^ the photo‐oxidation process proceeds to completion. The rate of PATP conversion on SiO_2_/TiO_2_ core/shell microbeads (T‐rex)^[18]^ was in between that of P25 nanoparticles and planar TiO_2_ thin films. (Figure S4). These data suggest that the morphology of supports is more important than the chemical nature of the material in controlling the photooxidation kinetics. To further confirm this hypothesis, two types of monodisperse SiO_2_ spheres, with average size of 235±1 nm and 6.47±0.32 μm were separately soaked in the same PATP solution. Upon drying, the Raman spectra were collected at the same irradiation power as a function of time. As shown in Figure 1d (see also S5), the kinetics of dimerization was remarkably different. The reaction proceeds on nanosphere supports, whereas is strongly inhibited in the case of the biggest, μm‐sized spheres.

**Figure 2:**
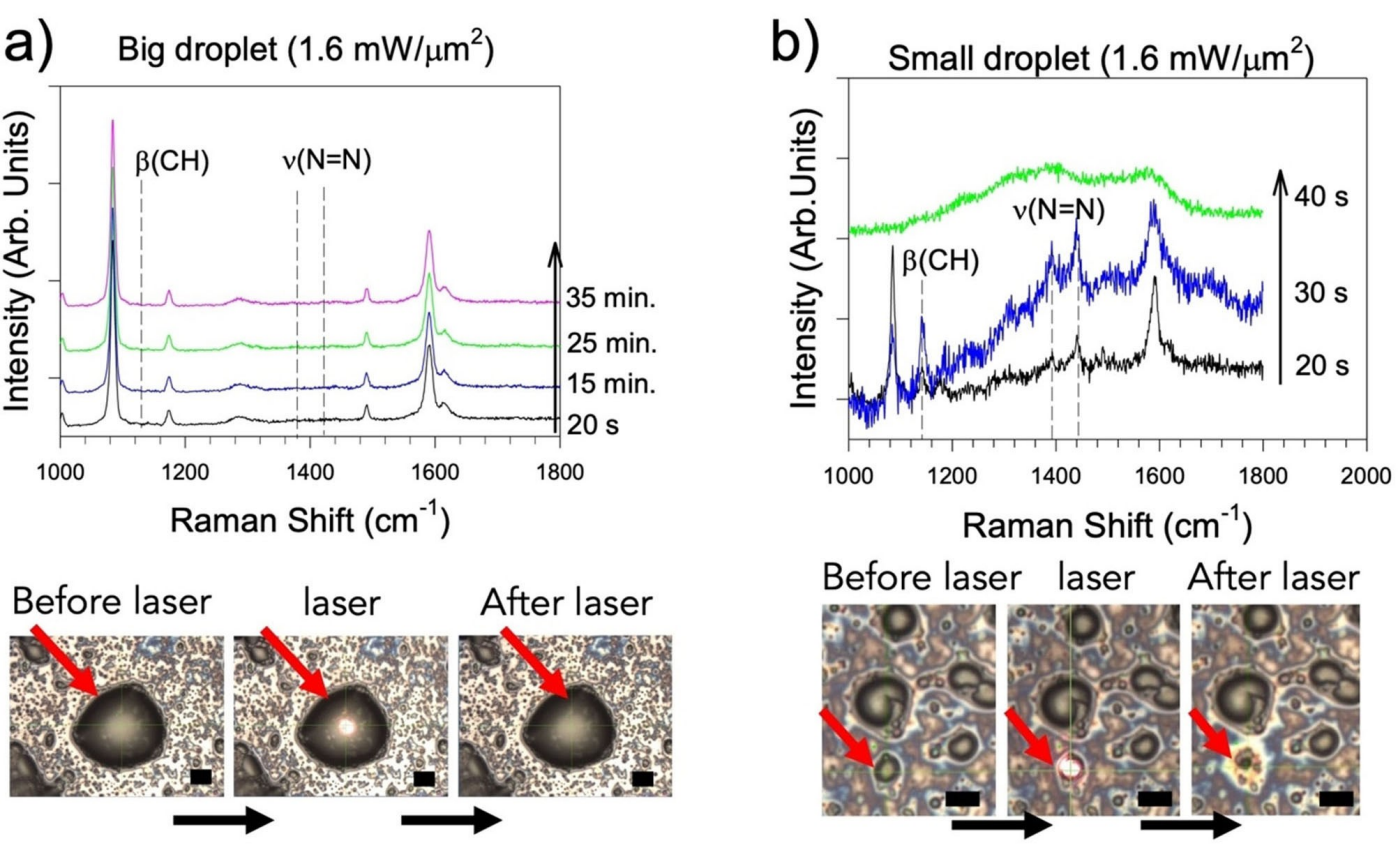
Spectral and morphological evolution of PATP photo‐oxidation induced by laser irradiation at 633 nm, 1.6 mW μm^−2^ for a) big (i.e. >30 μm) and b) small (i.e. <5 μm) droplets (concentration of the original PATP solution: 10^−4^ M). Each spectrum was acquired for 10 s at the intervals of time indicated in the labels. The optical microscope images below each graph show examples of the droplets utilized for these experiments. In the case of small droplets, the laser spot, shown in the central snapshot of panel b, fully overlaps the droplet diameter. Scale bar: 5 μm.

A further confirmation in this regard is provided by the series of experiments shown in Figure 2 and S6,7, in which droplets of PATP with a variety of different sizes were obtained by drying an aliquot (e.g. 5–10 μL) of the same ethanol solution on either glass or silicon substrates in the absence of TiO_2_ or any other mediators. Surprisingly, the irradiation of these PATP droplets at 633 nm gave different results depending on their size. Freshly deposited droplets with size bigger than 20–30 μm were stable, even for prolonged (up to 35 min.) irradiation at the maximum power (1.6 mW μm^−2^). No DMAB spectral modes were observed and no evidence of any modification in droplet morphology was found from optical microscopy inspection (Figure 2a). However, when their size is reduced to the 1–5 μm range, the droplets undergo a rapid oxidation that passes through dimerization and terminates with their complete carbonization, as shown in Raman spectra an optical microscope images (Figure 2b and S6). Although it is not possible to characterize the oxidation steps that follows the irradiation of DMAB, the rapid increase of the Raman background, particularly in the 1300–1800 cm^−1^ region, suggests the formation of intermediate carbonaceous species that contribute to the spectral fluorescence. Raman spectra of amorphous carbon were observed at the end of the oxidation (Figure 2b). When the irradiation power was reduced to 0.16 mW μm^−2^ the photo‐oxidation kinetics was significantly slowed down and the progress of DMAB formation could be monitored in more detail (Figure S7). One can argue that the dependence of the reaction kinetics on the droplet size could be a proof of the photothermal nature of the process. In this regard, heat would be better dissipated over larger amount of sample, whereas small droplets could be more efficiently heated.

However, control experiments showed that the Raman spectra of small PATP droplets remain unaltered when the sample is irradiated with a 785 nm laser, even though its power is remarkably higher (260 mW μm^−2^, Figure S8) than that of the 633 nm counterpart. This means that dimerization primarily depends on the wavelength of the exciting light source and pure thermal effects are not sufficient to drive dimerization.

Further experiments were performed on both PATP powders and solutions that have been heated in a Raman microscope cell from 25 to 125 °C. In situ Raman analysis was run using a 785 nm laser source, to avoid any direct laser‐induced azo‐coupling. Unfortunately, we cannot further extend the temperature range as the spectral background saturates the Raman signal for *T*>130 °C. We note that PATP is not thermally dimerized within this temperature range. As expected, the spectral background progressively increases as a function of heating, yet its shape is completely different from that of the samples irradiated at 633 nm and room temperature (Figure S9). The fact that the progressive increase of spectral background observed during photodimerization is not caused by purely thermal effects is also confirmed by the Raman spectra of TiO_2_ nanoparticles soaked in ethanol in the absence of PATP, which remain unaltered even for prolonged irradiation (Figure S10).

Thus, we can conclude that the mere increase of local temperature is not a determining factor for driving the PATP‐to‐DMAB transformation. As for the plasmonic counterparts, a possible clue to solve this conundrum is to consider the role of atmospheric oxygen. In fact, we observed that PATP droplets with sizes around 30–40 μm, which are unreactive under laser irradiation when freshly deposited, undergo a slow, yet progressive oxidation after being aged in air overnight (Figure S11). Several papers on plasmonic nanoparticle‐assisted PATP‐to‐DMAB conversion report that no dimerization products are formed when the reaction is carried out in the absence of oxygen, yet Zhao et al.^[12]^ predicted the possibility to obtain photodimerization without oxygen by means of hot holes and Yan et al. reported the formation of DMAB in N_2_ atmosphere by adding AgNO_3_ to Ag nanoparticles.^[8]^

The enthalpy of O_2_ bond is about 498 kJ mol^−1^, which prevents its direct dissociation under the experimental conditions utilized in this work. In fact, since the energy of each photon emitted at 633 nm is 3.15×10^−19^ J, the energy flow for a typical full power irradiation microRaman experiment is limited to 1.6 mJ μm^−2^ s^−1^. Moreover, the inertness of PATP irradiated in air at 785 nm and in situ thermal heating control experiments points out to a wavelength‐dependent photochemical activation of oxygen. This hypothesis was assessed through a series of confocal Raman microspectroscopy experiments carried out on PATP powders with and without atmospheric oxygen, which indicate that laser irradiation *per se* is not sufficient to trigger dimerization and confirms the key role of oxygen in the process (Figure S12). At the same time, these results suggest that oxygen can be involved in dimerization only if activated by laser irradiation. In the case of plasmon‐assisted photodimerization, plasmonic nanoparticles play a key role in activating atmospheric oxygen. In the model elaborated by Huang et al.^[14]^ laser irradiation induces direct electron transfer from the plasmonic nanoparticles to oxygen, producing O_2_^−^ species. The latter are strongly adsorbed on the surface of the nanoparticles and assists PATP photodimerization, leveraging on the rise of local temperature of metal due to ohmic losses. In the present case, the plasmonic assistance cannot be exploited. This means that the oxygen‐mediated PATP photodimerization should follow another pathway.

In this regard, we note that irradiation at 633 nm (1.96 eV) can directly promote the excitation of molecular oxygen to singlet oxygen states (O_2_ b^1^Σ_g_^+^ and O_2_ a^1^Δ_g_) with both monomol and dimol transitions.^[13]^

**Figure 3:**
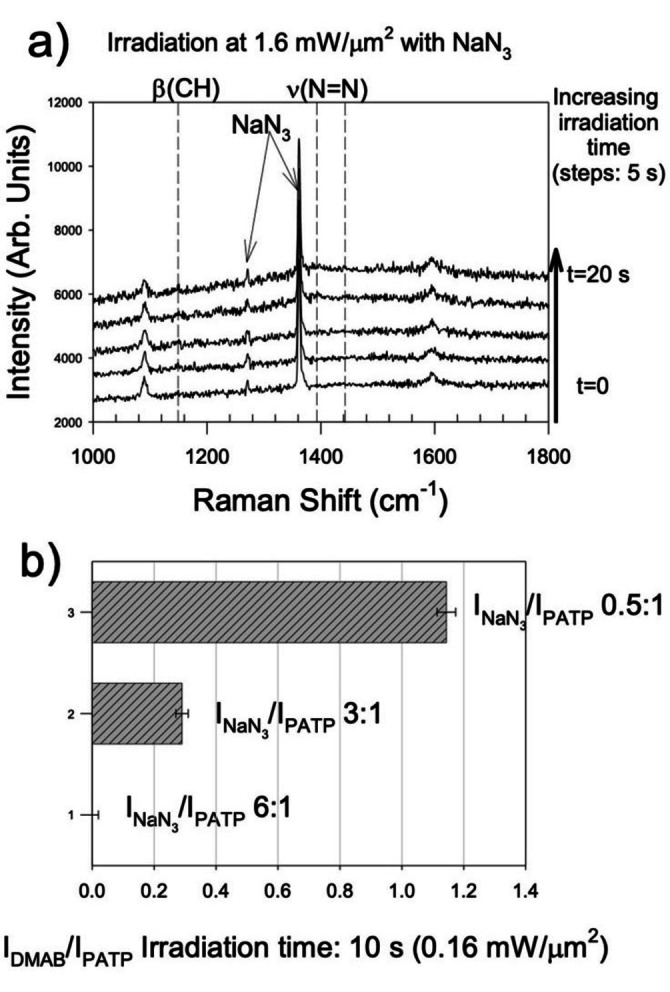
a) Spectral evolution showing the inhibition of photo‐oxidation of a PATP small bubble (10^−2^ M) under laser irradiation at 633 nm, 1.6 mW μm^−2^ in the presence of an excess of NaN_3_. Each spectrum was acquired for 1 s. In the absence of NaN_3_ this type of bubbles undergo complete oxidation under the same conditions (Figure S14); b) evolution of dimerization as a function of the relative abundance of NaN_3_. The extent of photodimerization was evaluated from the relative intensity ratio between the Raman mode of DMAB at 1142 cm^−1^ and that of PATP at 1087 cm^−1^. The relative abundance of NaN_3_ was evaluated by the ratio between the intensity of the NaN_3_ mode at 1360 cm^−1^ and that of PATP mode at 1087 cm^−1^.

**Figure 4:**
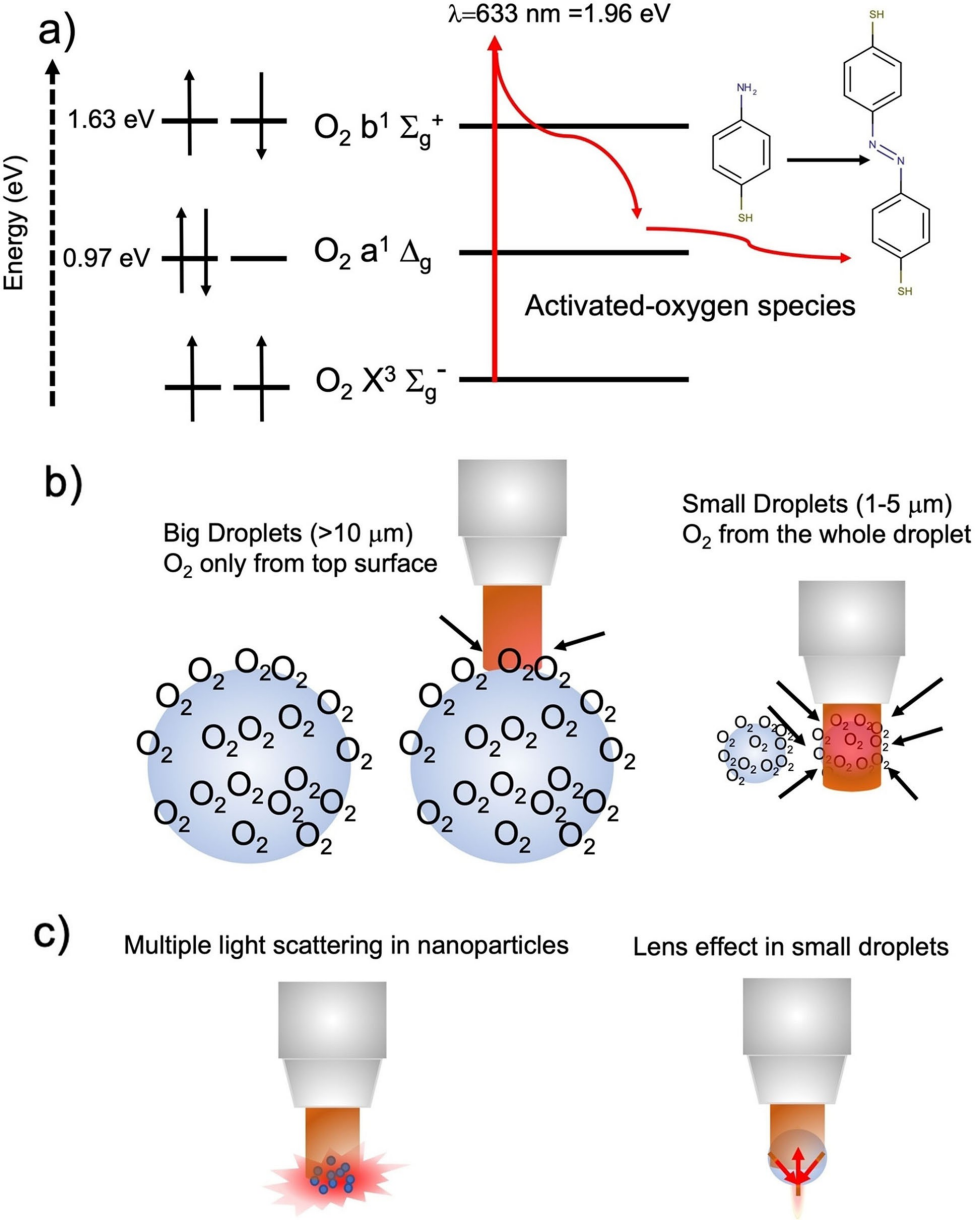
a) Scheme of the model of plasmon‐free photo‐oxidation of PATP. The He−Ne laser (λ=633 nm, 1.96 eV) can directly excite the transition of O_2_ from triplet to a singlet state O_2_ b^1^∑_g_^+^, which eventually decays to a O_2_ a^1^Δg state. Both types of singlet states can concur in promoting the photo‐oxidation of PATP. b) Effect of droplet size: In the case of droplets significantly bigger than the laser beam size only O_2_ molecules adsorbed on the top surface can be excited, whereas irradiation of small droplets involves almost the whole sphere surface, which is about 4 times larger, making photo‐oxidation more efficient. c) Possible additional optical effects that might contribute to increase the photooxidation kinetics. In the case of nanoparticles (as for P25‐TiO_2_ and ZnO nanopowders or SiO_2_‐235 nm spheres discussed above) multiple scattering of light could extended the optical pathlength of the laser beam, stimulating the activation of O_2_ species adsorbed on the surface. PATP droplets with size compared to that of the laser beam can play as micro‐lenses, concentrating the local electromagnetic field. See the main text for comments.

In monomol transition a single ground‐state O_2_ molecule (^3^O_2_) can be photoexcited to either O_2_ b^1^Σ_g_^+^ (highest energy) or O_2_ a^1^Δ_g_ (lowest energy) states. If the photon energy is above 1.62 eV the O_2_ b^1^Σ_g_^+^level can be reached, which undergoes rapid de‐excitation to the O_2_ a^1^Δ_g_ state. In parallel, dimol transitions can be stimulated at 633 nm as well. In this type of transitions, whose absorption coefficient is very low, one photon is absorbed by two ground state oxygen molecules linked in a transient complex [^3^O_2_–^3^O_2_], producing two singlet oxygen species. In any case, singlet oxygen species are strongly oxidating agents that can easily promote the generation of oxygen radical species. The latter can trigger and sustain the oxidation of PATP into carbonaceous species, which passes through the formation of DMAB. The role of singlet oxygen was assessed by mixing the PATP solution with sodium azide (NaN_3_), which is normally utilized as a quencher for singlet oxygen.^[13]^ Interestingly, the presence and relative abundance of NaN_3_ can be directly monitored from the symmetric stretching of the azide group at about 1360 cm^−1^ (see also the Raman spectrum of the sodium azide reference in S13). Figure 3a shows that NaN_3_ can completely prevent photodimerization, even at the highest irradiation power. In general, the extent of the reaction, which was measured from the intensity ratio of DMAB_1142_/PATP_1087_ modes, is inversely dependent on the amount of NaN_3_ (Figure 3b). These experiments demonstrate that laser‐induced photodimerization passes through the excitation of singlet oxygen, which is a strong oxidant (Figure 4a).

The progress of reaction through activated oxygen species could also explain the observation of different reaction kinetics for differently sized PATP droplets.

As schematically illustrated in Figure 4b, small droplets are probably more reactive than their bigger counterparts because they are commensurate to the laser beam size, which ranges between 1.5 and 2.12 μm. This allows for a direct excitation of almost all O_2_ molecules physisorbed all around the droplet surface. As the laser excitation is continuous wave‐type, photons are constantly supplied, and local power becomes a critical parameter to control the extent of photo‐oxidation over time.

On the other hand, in the case of bigger droplets this excitation is limited to the portion of surface defined by the beam size. In this case the amount of O_2_ molecules is not sufficient to activate and extend oxidation above the detection limit. On the other hand, when aged in air for several hours, bigger droplets are allowed to adsorb more oxygen, thus the probability of generating oxidizing species by photoactivation is increased. Similarly, the Raman experiments on TiO_2_ nanoparticles, microspheres and thin films, as well as those with differently sized SiO_2_ spheres can be interpreted according to this model. The higher the sample surface area, the higher O_2_ adsorption and the faster the photo‐oxidation. In addition to the extended availability of surface oxygen species, nanoparticles and small droplets may also take advantage of an increased optical pathlength for laser (Figure 4 c). Nanoparticles and colloids are indeed well‐known to promote surface light scattering,^[20]^ which could maximize the interaction of laser with oxygen and PAPT molecules. Moreover, likewise micron‐sized spherical colloids,^[19]^ small droplets could play as secondary micro lenses, promoting the local concentration of electromagnetic field, which could be beneficial for driving the oxidation process. The role of morphology in light management at the nanoscale has been widely demonstrated,[^18^, ^20^] however the quantification of its contribution in this type of reactions deserves further investigation and will be addressed in future studies.

These results are also useful for interpreting some contradictory results reported in studies on plasmonic nanoparticles. For example, the fact that no dimerization was observed in some previous reports^[7]^ upon irradiation at 633 nm for short time (1 s), even in the presence of plasmonic nanoparticles, may depend on the fact that in aqueous solution the concentration of dissolved oxygen is below 0.27 mM, which makes the generation of oxygen photo‐excited species quite inefficient. Other works reported indeed the occurrence of photodimerization at 633 nm in air and the role of oxygen species photoactivated with the assistance of hot electrons coming from laser excitation of plasmonic nanoparticles has been demonstrated.[^9^, ^14^, ^15^, ^16^] Hot electrons are involved also in the photoreduction of PNTP to PATP, as clearly demonstrated in previous works.[^21^, ^22^] In that case the presence of plasmonic metals is necessary to drive photoreduction to PATP. The fact that hot electrons are utilized to explain the opposite, photo‐oxidation process, is interesting. The present work demonstrates the existence of an alternative pathway, based on direct activation of singlet oxygen, which is a strong oxidating species. The key point of novelty is the discovery that PATP can be transformed by photoactivated oxygen even in the absence of any plasmonic mediators. This means that hot carriers may be helpful to assist the generation of reactive oxygen species (^1^O_2_ and derivatives) but not necessary to drive a process that has been normally considered the paradigm of plasmon‐driven reactions. The beneficial role of nanoparticles could be not only limited to boost the generation of reactive oxygen species, but it could be associated to molecule binding and high surface areas. Plasmonic nanoparticles provide an excellent surface to bind thiol groups, which constrains the PATP molecules to form self‐assembled monolayers. Although often neglected, this regular proximity could play a key role in promoting dimerization. On the other hand, the formation of disulfide bridges and hydrogen bonds hamper photodimerization, thus films made from highly concentrated solutions or powdered samples are relatively inert. This work also shows that even non‐plasmonic nanoparticles can be helpful for improving the kinetics of photo‐oxidation and suggests the possibility to exploit nano‐optical effects to control the interactions of laser with the adsorbates.

## Conclusion

Overall, this work widens the horizons of light‐driven surface reactions, showing that oxygen photoactivation, which is involved in a variety of chemical and biochemical processes, can be achieved and controlled by morphology factors, without need of any charge‐transfer mediators, such as plasmonic nanoparticles. It also provides new clues to interpret Raman and SERS spectra characterized by low temporal stability and ill‐defined “chemical enhancement”.^[17]^ Many SERS data cannot be replicated in the same region of the substrate, which limits their analytical usefulness. More generally, in many cases the Raman analysis of soft matter is affected by sample damaging that is usually ascribed to local heating. This forces the experimentalist to run spectral acquisition at reduced laser power, which in turn reduces the intensity of the Raman signal. This results in lower sensitivity, which is often detrimental for both analysis and sensing. In the case of SERS experiments, the need of attenuating the power of incoming laser often negatively counterbalances the high local concentration of the electromagnetic field generated by plasmonic substrates. Moreover, the fact that most of SERS spectra show the well‐known “cathedral peaks”, characteristic of the formation of carbonaceous species, in the 1300–1600 cm^−1^ region, indicates that photo‐oxidation is a common interference to SERS analysis.^[23]^ As demonstrated in this work for PATP, if singlet oxygen had a role in laser‐induced oxidation of these molecules, its activation could be inhibited by adding quenchers, without need of reducing laser power.

In perspective, the study of laser‐induced production of oxygen photoactivated species, so far limited to photobiology and phototherapy, may open new frontiers for surface photochemistry, with a variety of applications in photocatalysis, environmental remediation and production of energy vectors from renewable sources.^[24]^

Experimental details and control experiments are reported in Supporting Information.

## Conflict of interest

The authors declare no conflict of interest.

1

## Supporting information

As a service to our authors and readers, this journal provides supporting information supplied by the authors. Such materials are peer reviewed and may be re‐organized for online delivery, but are not copy‐edited or typeset. Technical support issues arising from supporting information (other than missing files) should be addressed to the authors.

Supporting Information

## Data Availability

The data that support the findings of this study are available from the corresponding author upon reasonable request.
