# Supplementary material for: 4‐Aminothiophenol Photodimerization Without Plasmons
Source: Angew Chem Int Ed Engl. 2022 May 19;61(28):e202205013. doi: 10.1002/anie.202205013 (PMC9401036; doi:10.1002/anie.202205013)
Supplement: Supplementary file 1 — Supporting Information [file ANIE-61-0-s001.pdf]

Angewandte Chemie
Eine Zeitschrift der Gesellschaft Deutscher Chemiker

Supporting Information

4-Aminothiophenol Photodimerization Without Plasmons

*I. Alessandri**

SUPPORTING INFORMATION

Table of Contents

S0. Experimental Details

S1-S5. Control experiments on different types of TiO_2 supports

S6. Additional Raman spectra of Fig 1d

S7-S10. Control experiments on PATP droplets

S11. Control experiments on P25 TiO_2 nanoparticles in the absence of PATP

S12. Confocal Raman experiments of PATP powders with and without oxygen

S13. NaN_3 Raman reference spectrum

S14. Control experiment without NaN_3

S0. Experimental Details

Chemicals

4-aminothiophenol powder (purity >97%), ethanol (99.8%) and sodium azide (>99.5%) were purchased by Merck-Sigma-Aldrich and utilized without any further purification.

The technical specifications of commercial P25-TiO_2 Aeroxide® nanopowders (Evonik) can be found in the online version of material datasheet.[1] Commercial ZnO nanopowders (average size<100 nm) were purchased by Merck-Sigma-Aldrich. The monodisperse nano- (average size 235±1 nm) and micro-(average size: 6.47±32 μm) spheres were purchased by Microparticles GmbH.

The SiO_2/TiO_2 core/shell microspheres (T-rex) were fabricated by following the experimental protocol reported in ref. 2.[2] The planar thin films were achieved with the same procedure, without using silica spheres.

Confocal microRaman experiments

The confocal microRaman experiments were carried out by a high-resolution Raman microscope (Labram HR-800, Horiba/Jobin-Yvon), equipped a Syncerity CCD detector and an Olympus B-41 microscope stage, with 4 optical objectives (10X, 50X, 50X Long Working Distance, LWD and 100X). The optical images were acquired directly from the Raman microscope stage. The Raman spectra were acquired with a 50X LWD objective (Numerical Aperture, N. A. 0.50). The *in-situ*, temperature Raman measurements were carried out with a Linkam HFS-91 thermal stage, using the same optical objective. This stage was also utilized for the measurements in the absence of oxygen. The anoxic conditions were obtained by saturating the chamber with pure nitrogen. Confocal analyses to differentiate surface and interior of the PATP crystals were carried out by progressively changing the focal plane through the crystals under analysis.

At least 10 different regions were analyzed for each sample tested in Raman experiments.

NaN_3 was utilized as a singlet oxygen quencher. 7 milligrams of NaN_3 in methanol were dissolved in 100 μL of of a 10^{-2} M solution of PATP and the final mixture was dropped on a silicon substrate for

Raman characterization. The same experiments were repeated by reducing the amount of NaN_3 by a factor of 2 and 10, respectively.

References:

1) P25-TiO_2 Aeroxide® datasheet available at products-re.evonik.com

2) Alessandri, I. Enhancing Raman Scattering without Plasmons: Unprecedented Sensitivity Achieved by TiO_2 Shell-Based Resonators *J. Am. Chem. Soc.* **2013**, 135, 5541-5544

S1. Control Experiment 1: PATP on P25-TiO_2 irradiated at 1.6 mW/μm^2

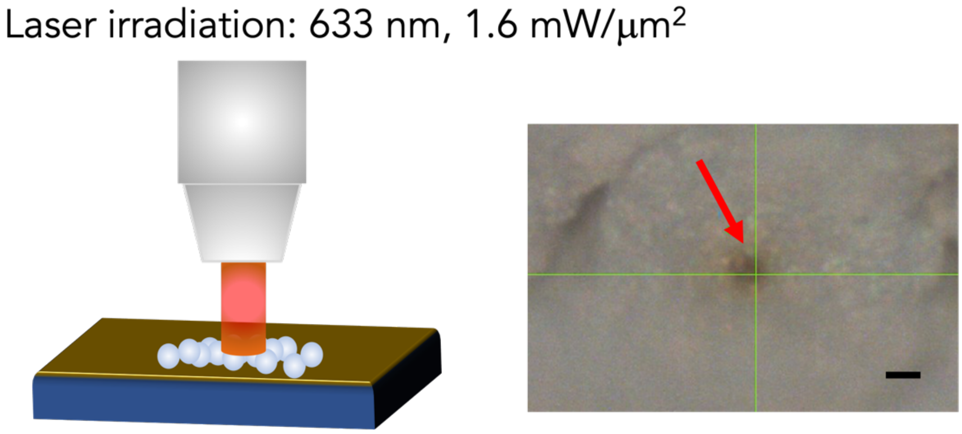

Figure S1. Optical microscope image showing the effects of He-Ne (λ=633 nm) laser irradiation at 1.6 mW/μm^2 on P25 nanopowders previously soaked in a 10^{-4} M PATP ethanol solution. The dark dot indicated by the red arrow shows the laser footprint. Scale bar: 5 μm.

S2. Control Experiment 2: PATP on P25-TiO_2 irradiated at 16 μW/μm^2

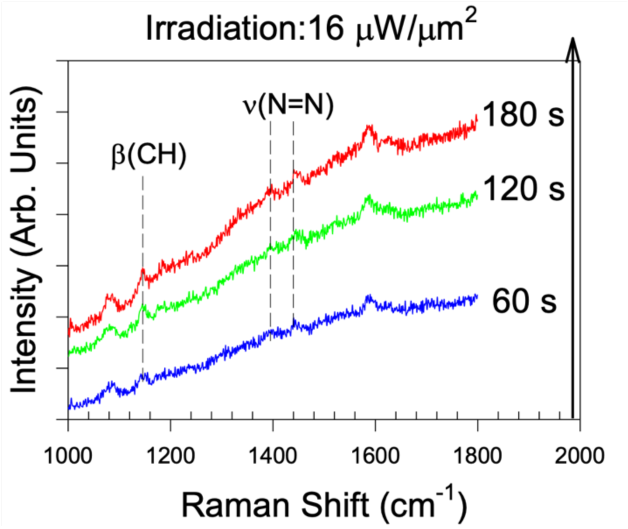

Figure S2. Raman spectra of P25 TiO_2 nanopowders previously soaked in a 10^{-4} M PATP ethanol solution, irradiated at 633 nm, 16 μW/μm^2). The Raman spectra have been stacked for clarity.

S3. Control Experiment 3: PATP on planar TiO_2 irradiated at 0.16 mW/μm²

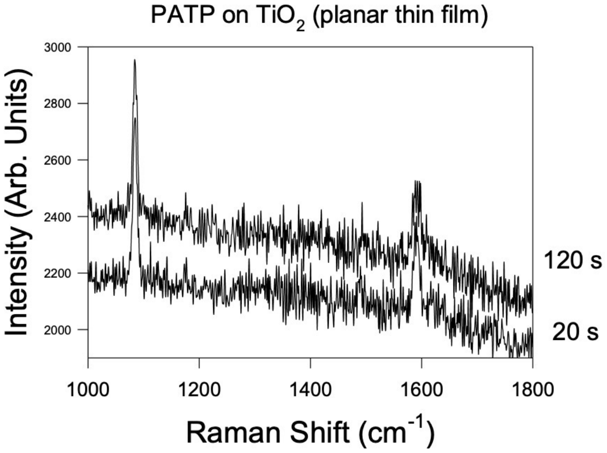

Figure S3. Raman spectra of anatase TiO_2 planar thin films previously soaked in a PATP 10^{-4} M ethanol solution irradiated at 633 nm, 0.16 mW/μm²). The Raman spectra have been stacked for clarity.

S4. Control Experiment 4: Photo-oxidation of PATP on planar T-rex beads.

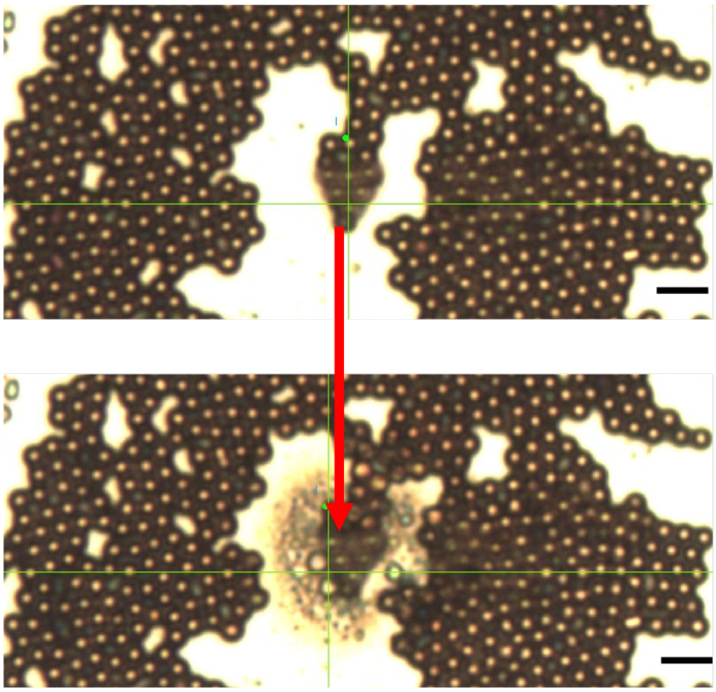

Figure S4. Optical microscope images showing the effects of 633 nm laser irradiation (1.6 mW/μm²) on SiO_2/TiO_2 core/shell microspheres (T-rex beads). Scale bars: 5 μm.

S5. Control Experiment 5: Complete sequence of spectra of Figure 1d.

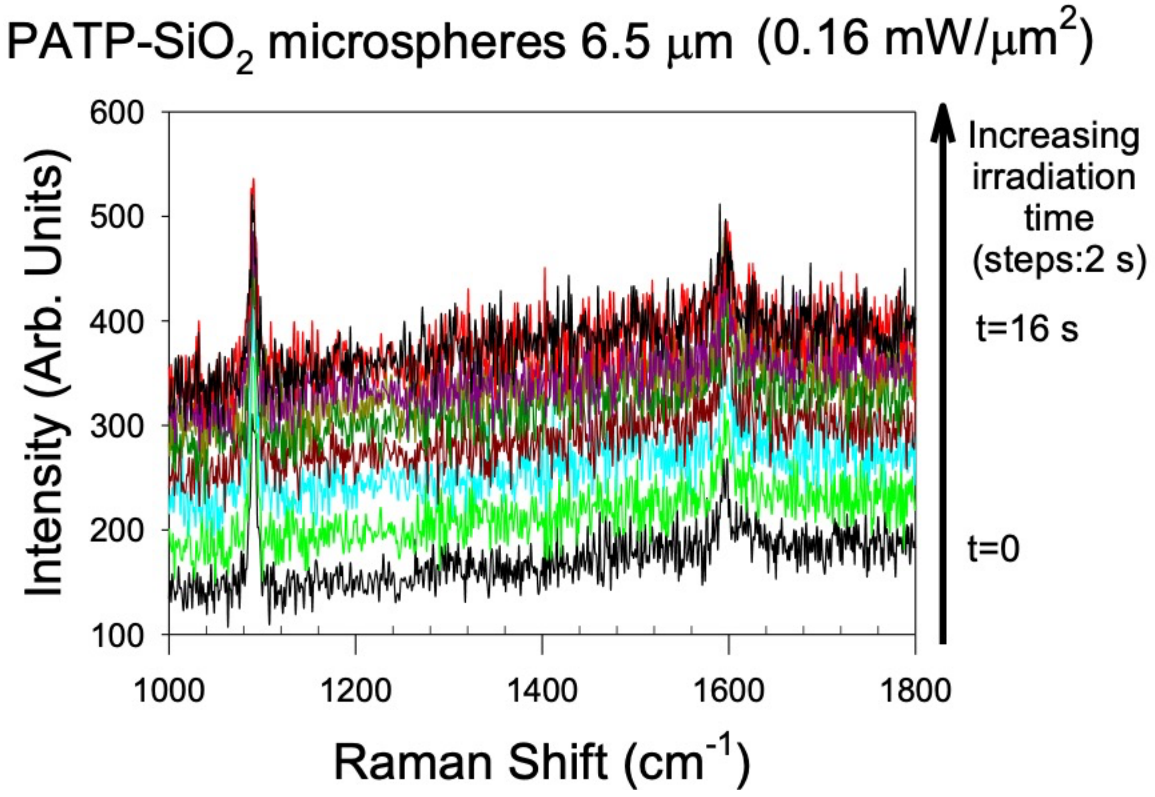

Figure S5. Sequence of Raman spectra of PATP supported on 6.5 micron-sized SiO_2 microspheres irradiated at 633 nm for 1 s. Spectra from 0 to 16 s of total irradiation are shown at increasing steps of 2 s. In figure 1d of the main text only spectra at t=0 and 16 s were shown for clarity.

S6. Control Experiment 6: Effect of laser irradiation on small PATP droplets

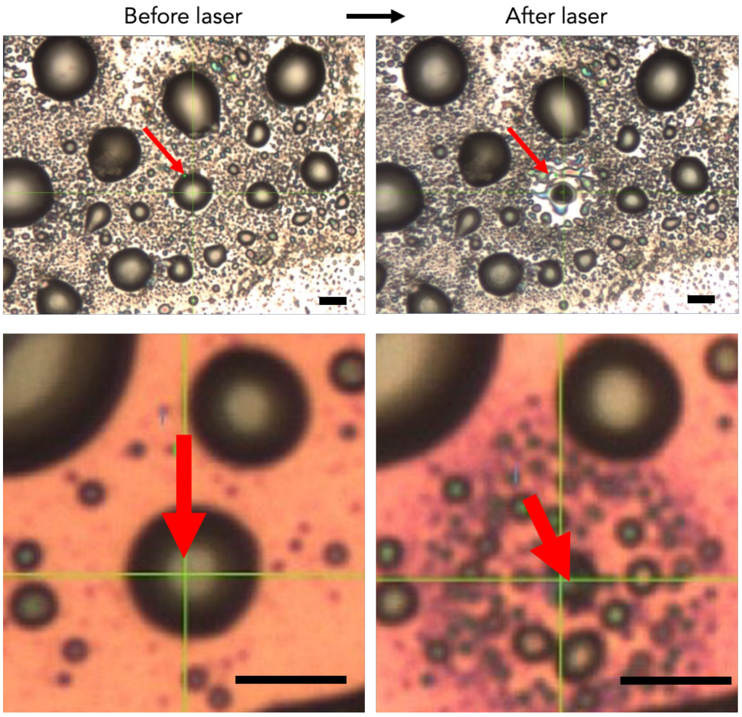

Figure S6. Optical microscope images showing examples of the effects of 633 nm laser irradiation (1.6 mW/μm^2) on PATP small droplets. Scale bars: 5 μm.

S7. Control Experiment 7: Raman spectra of big and small PATP droplets irradiated at 0.16 mW/μm²

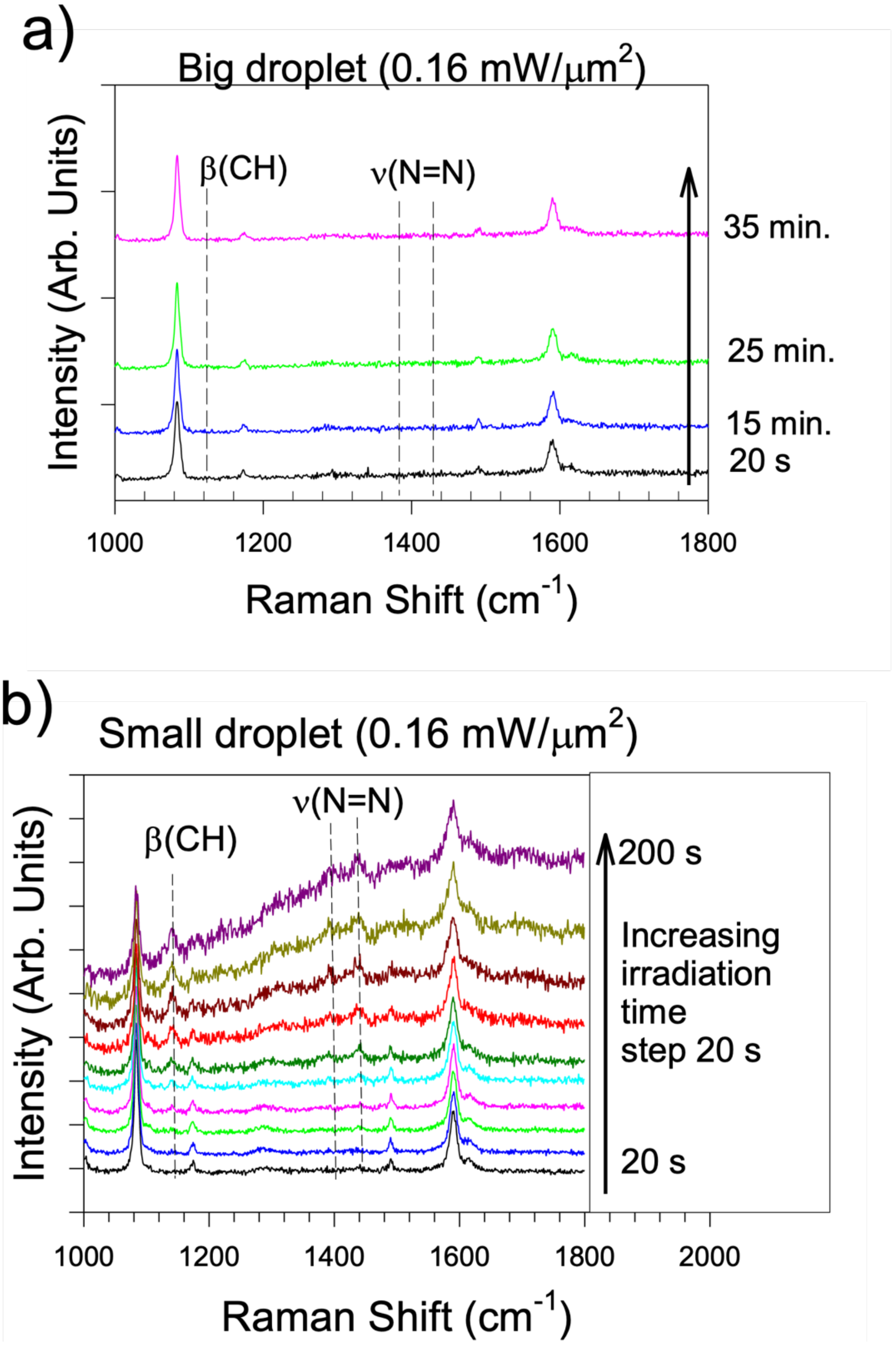

Figure S7. Temporal evolution of the PATP (and DMAB) Raman spectra for a) big (*i.e.* >30 μm) and small (*i.e.* <5 μm) droplets, irradiated at 0.16 mW/μm².

S8. Control Experiment 8: Effect of prolonged irradiation at λ=785 nm on small PATP droplets

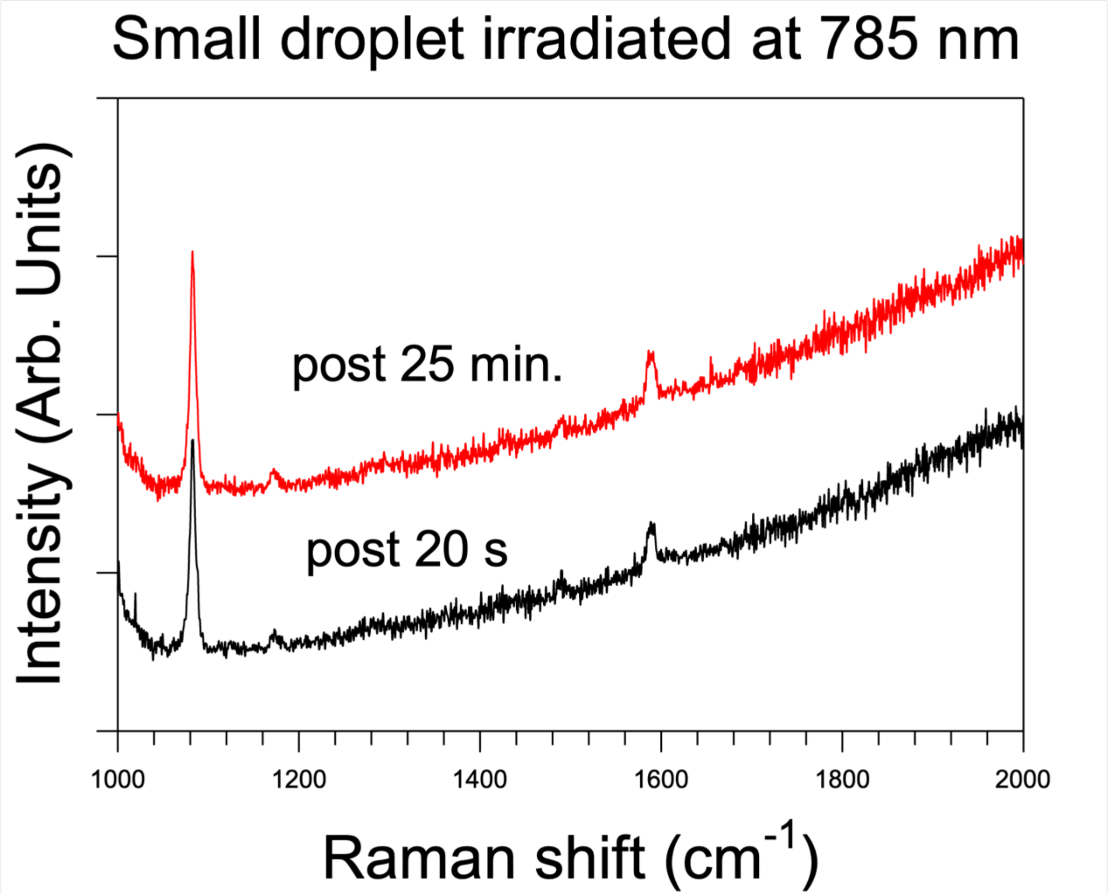

Figure S8. Raman spectra of PATP small (< 5 μm) droplets irradiated at 785 nm (260 mW/μm^2) at different exposure time. The spectra were stacked for clarity.

S9. Control Experiment 9: In situ thermal heating experiments

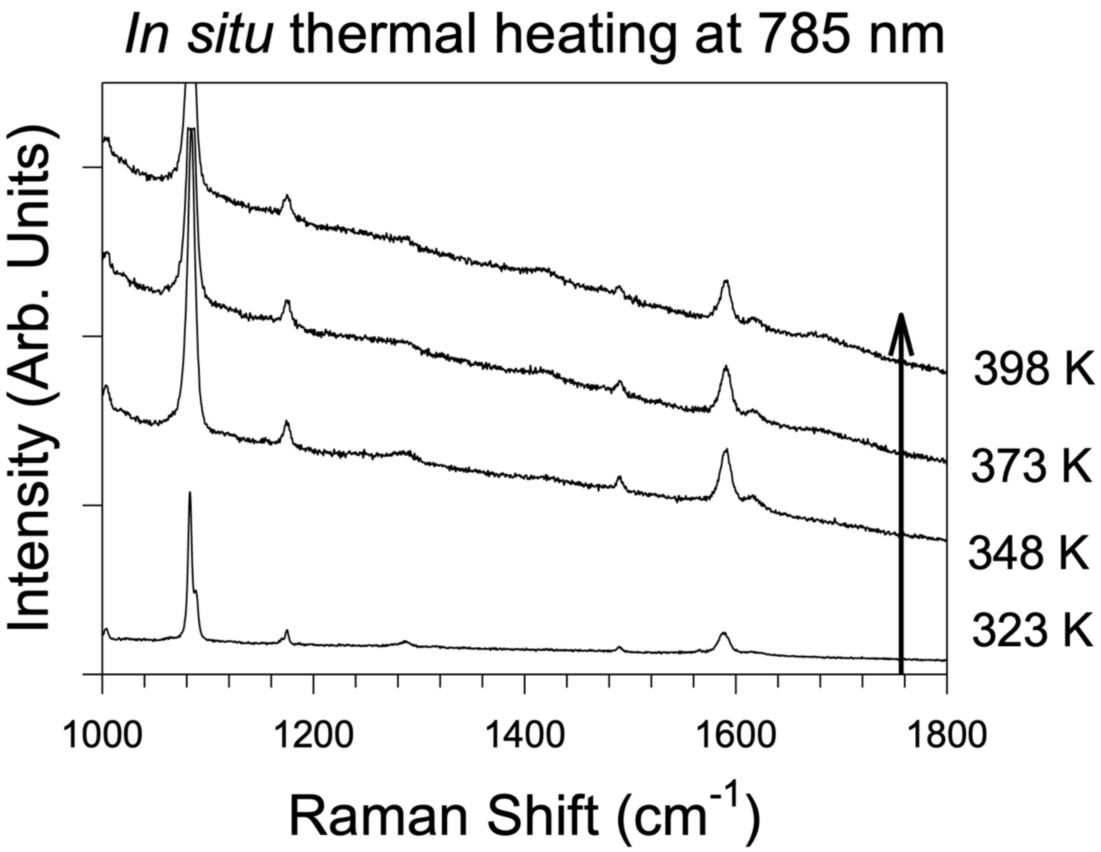

Figure S9. *In-situ* thermal heating of PATP powders irradiated at 785 nm in a Linkam cell. The acquisition time for each scan was 10 s.

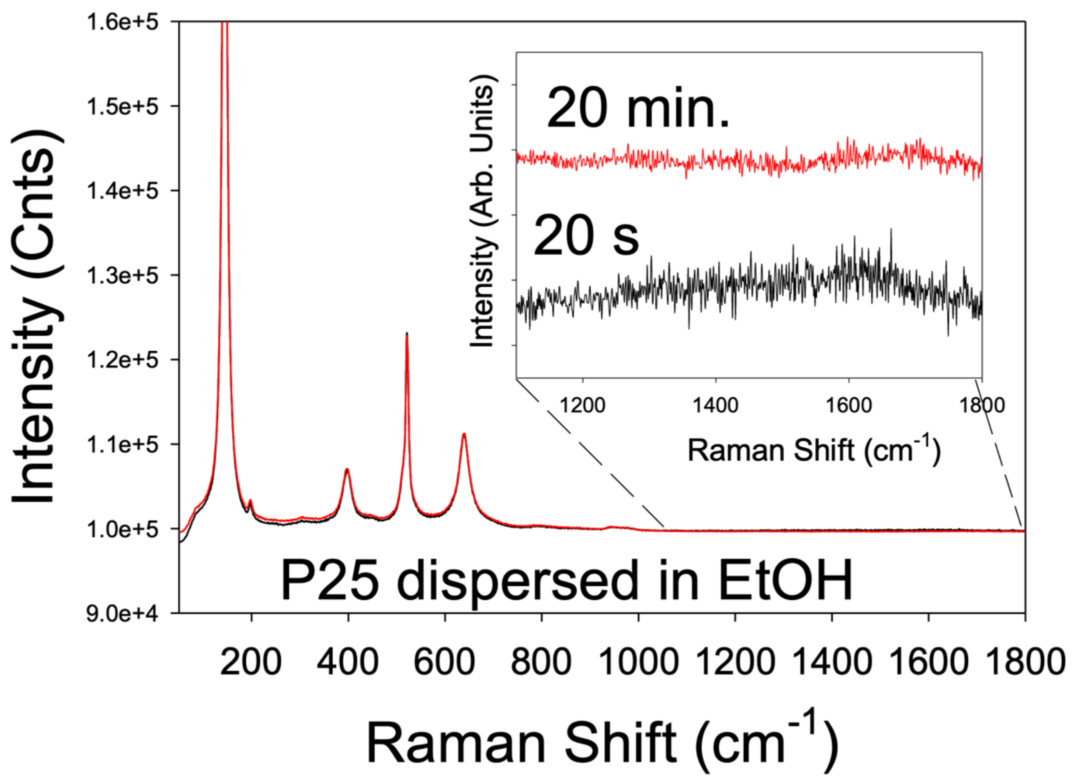

Figure S10. Raman spectrum of P25 soaked in ethanol in the absence of PATP and irradiated under the same conditions (633 nm, 0.16 mW/µm^2) of experiment shown in Figure 1 (main text). The Raman spectra have been stacked for clarity. The inset shows a zoomed view of the 1000-1800 cm^{-1} region.

S11. Control Experiment 11: Raman spectra of big PATP droplets aged in air overnight under laser irradiation at 1.6 μW/mm².

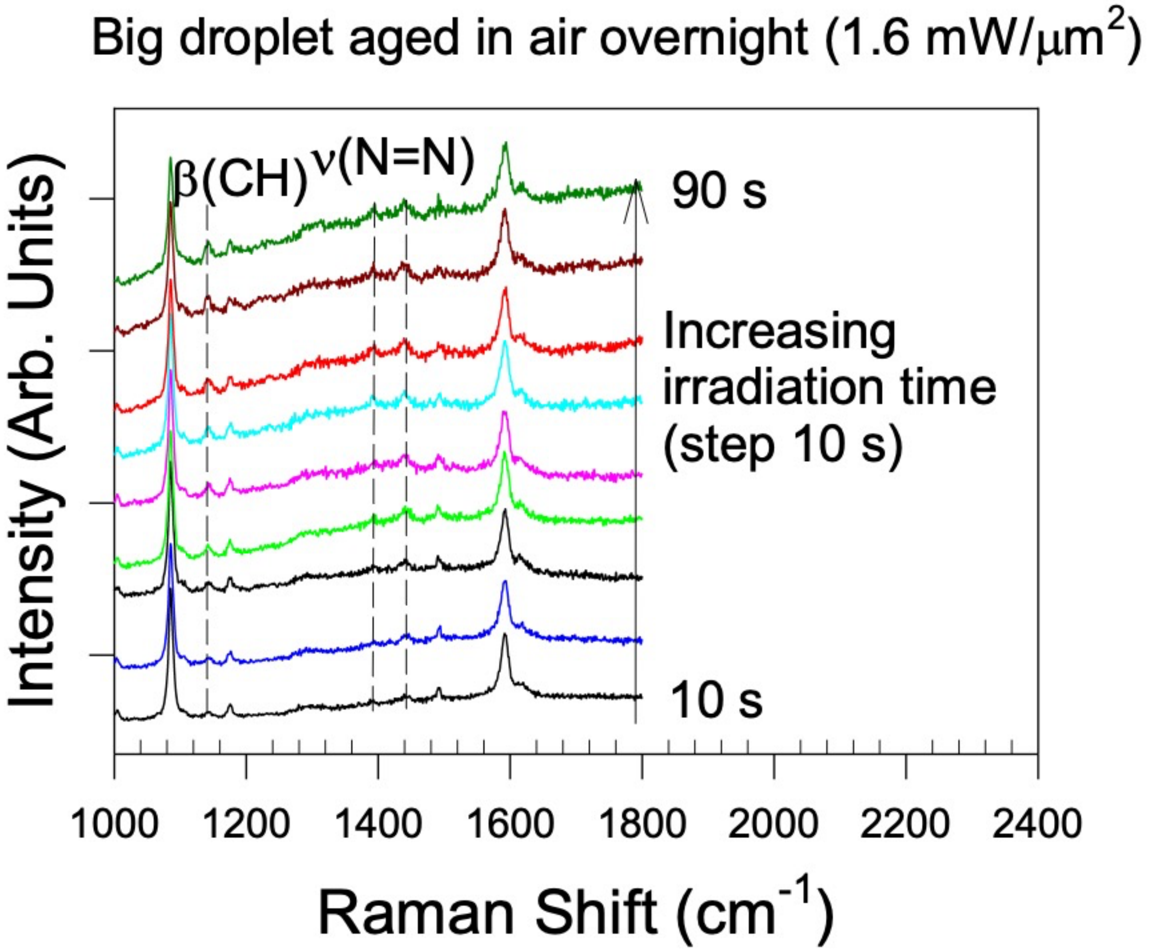

Figure S11. Temporal evolution of the PATP (and DMAB) Raman spectra for big (*i.e.* >30 μm) droplets aged overnight, irradiated at 1.6 mW/μm².

S12. Control experiment 12: Confocal Raman experiments with and without oxygen

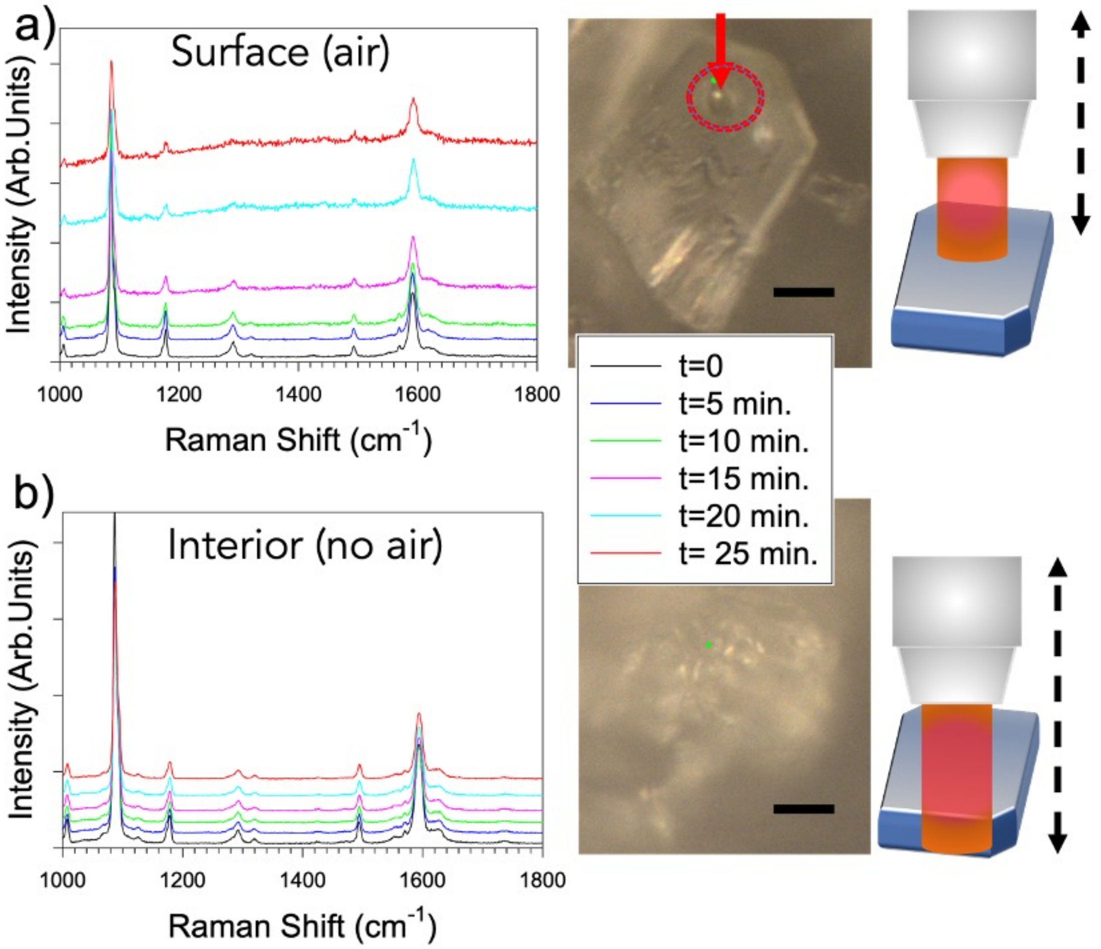

Figure S12. Temporal evolution of the Raman spectra of PAPT powders irradiated at 633 nm, 1.6 mW/μm^2 on a) surface and b) inner (bulk) regions of the crystals, obtained by confocal microscopy acquisition at different penetration depth (see the scheme on the right). The spectra acquired from the inner regions of the samples are analogous to those acquired in nitrogen-saturated atmosphere. The corresponding optical microscope images are shown (scale bar: 5 μm). In the case of surface focusing (Panel a), the formation of the laser footprint is indicated by the red circle. No morphological modification was observed either for interior regions or oxygen-free surfaces (Panel b).

S13. Control Experiment 13: Raman spectrum of NaN_3 powder.

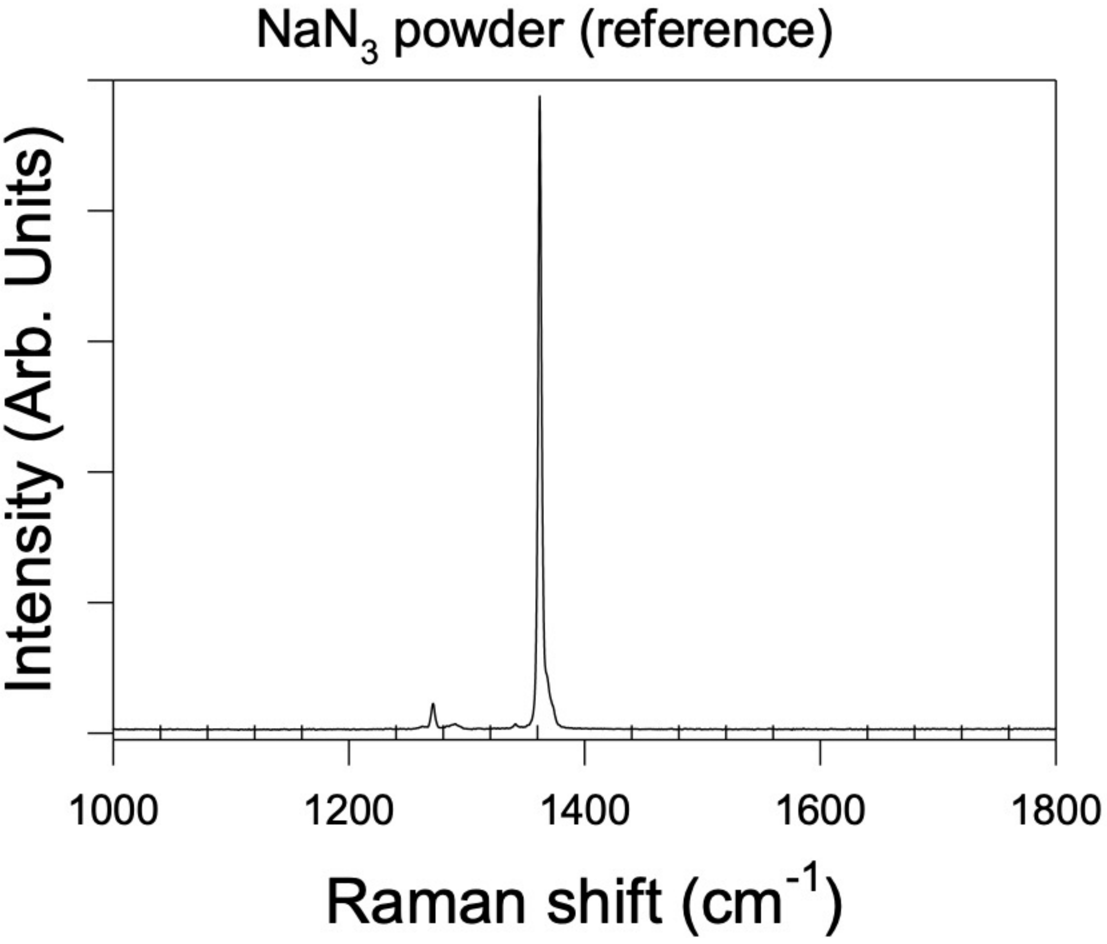

Figure S13. Raman spectrum of NaN_3 powder (reference).

S14. Control Experiment 14: Effects of irradiation at 1.6 mW/μm^2 on small bubbles without NaN_3

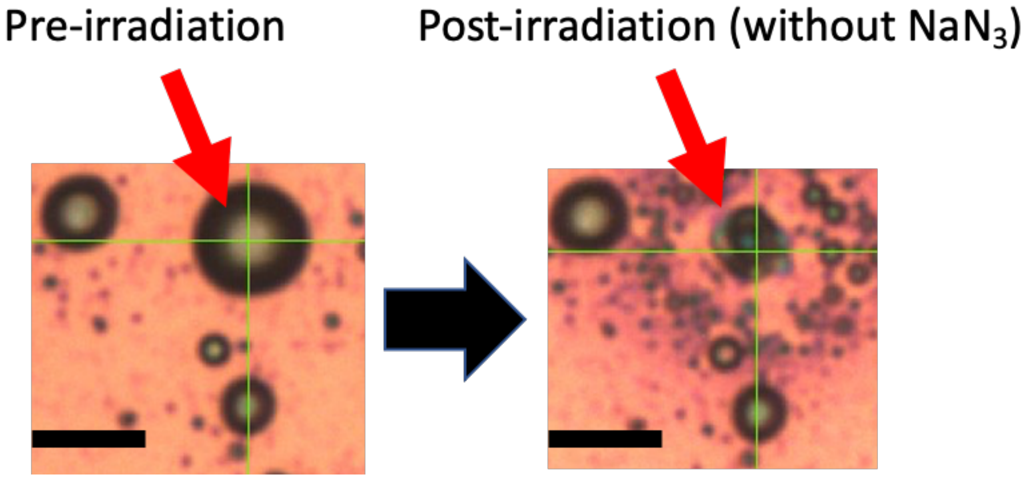

Figure S14. Effect of irradiation at 1.6 mW/μm^2 on a PATP small bubble (Concentration of the original solution: 10^{-2}M) in the absence of NaN_3. Scale bars: 5 μm.
